# Protective Effects of Yinchenhao Decoction on Cholesterol Gallstone in Mice Fed a Lithogenic Diet by Regulating LXR, CYP7A1, CYP7B1, and HMGCR Pathways

**DOI:** 10.1155/2018/8134918

**Published:** 2018-09-17

**Authors:** Yong Meng, Ke Meng, Xin Zhao, Donghua Li, Qiaoying Gao, Shangwei Wu, Yunfeng Cui

**Affiliations:** ^1^Tianjin Medical University, 22 Qixiangtai Road, Heping District, Tianjin 300070, China; ^2^Institute of Acute Abdomen in Integrative Medicine, Tianjin Nankai Hospital, Nankai Clinical School of Medicine, Tianjin Medical University, 122 Sanwei Road, Nankai District, Tianjin 300100, China; ^3^Department of Surgery, Tianjin Nankai Hospital, Nankai Clinical School of Medicine, Tianjin Medical University, 122 Sanwei Road, Nankai District, Tianjin 300100, China

## Abstract

The study attempted to elucidate whether lipid genes are closely associated with lipid metabolic abnormalities during the lithogenic time and how Yinchenhao Decoction (YCHD) works on the transcriptions of lipid genes against cholesterol gallstone model. C57BL/6J mice fed on lithogenic diet (LD) were used for model establishment and randomized into 5 groups. All groups received LD for different weeks with isometrically intragastric administration of YCHD or NS. Biochemical tests were measured and liver tissues were harvested for histological and genetic detection. It was found that all groups with increasing LD showed a following tendency of gallstone incidence, bile cholesterol, phospholipids, total bile acid, and cholesterol saturation index (CSI). Conversely, YCHD could significantly normalize the levels of gallstone incidence, bile lipids, and CSI (CSI<1). As lithogenic time progressed, ABCG5, ABCG8, PPAR-*α*, and ABCB4 were upregulated, and SREBP2, CYP7A1, and CYP7B1 were downregulated, while CYP7A1, CYP7B1, LXR, and HMGCR mRNA were increased 3-fold under the administration of YCHD. It was concluded that abnormal expressions of the mentioned genes may eventually progress to cholesterol gallstone. CYP7A1, CYP7B1, LXR, and HMGCR mRNA may be efficient targets of YCHD, which may be a preventive drug to reverse liver injury, normalize bile lipids, facilitate gallstone dissolution, and attenuate gallstone formation.

## 1. Introduction

Gallstone disease is one of the most common gastrointestinal disorders encountered in clinical practice [1]. The incidence of gallstone disease is racially diverse. The disease has prevalence as high as 5.9% to 21.9% in the developed countries and 3.1% to 10.7% in Asia, while the incidence is 4.21% to 11.0% in China [2, 3]. Chief among them is the cholesterol gallstone which accounts for nearly 70% of gallstones and mainly located in gallbladder [4, 5]. From the perspective of pathogenesis, the stones are primarily ascribed to excessive cholesterol accumulation in bile, gallbladder hypomotility, and gastrointestinal dysfunction [6, 7]. Most patients with cholecystolithiasis have no obvious clinical symptoms. 40% of patients have clinical symptoms and severe complications after 40 years of age and need to be operated on [8]. In recent years, laparoscopic cholecystectomy (LC) has gained widespread acceptance and become a gold standard for the treatment of cholelithiasis [9]. Quick recovery, clear curative effects, and fewer complications are advantages of LC comparing with the conventional opening surgery. However, the incidence of biliary injury during LC is 0.39%, which can be long lasting morbidity and fatal to the patients. They may also increase the cost involved in treatment and result in medical litigation. Researchers believe that complex complications should be better avoided than treated [10]. In view of the above conditions, the most direct way to avoid the occurrence and recurrence of cholelithiasis from gallstone is to prevent its formation in advance, which meets the concept of “preventive treatment of disease” in Traditional Chinese Medicine (TCM). Thus, prevention of the formation and recurrence of gallstones is essential. Nowadays, more and more attention has been paid to the studies of the treatment and prevention of gallstone by TCM. For example, the study by Xiao Wu et al. examined the mechanism of Lidan Granule (LDG) using a guinea pig model and showed that a promising effect from LDG can ameliorate the effect of a lithogenic diet on the hypothalamic-pituitary-adrenal (HPA) axis and bile components [11]. Therefore, TCM with long history and collected clinical experience should be attached more importance for its potential in disease prevention and health promotion.

Yinchenhao Decoction (YCHD), named Inchinko-to or TJ135 in Japan is one of the TCM remedies initially recorded in “Shanghanlun”, dating back to thousands of years ago. This classical recipe is made up of three principal ingredients: Herba Artemisiae Scopariae (Yinchen), Fructus Gardeniae (Zhizi), and Rheum rhabarbarum (Da Huang) at the weight ratio of 10: 5: 3 [12, 13].

As is known to us, TCM has specific drug compatibility consisting of four elements: the monarch drug (Jun), minister drug (Chen), assistant drug (Zuo), and servant drug (Shi). That is to say, in the disassembled prescription of YCHD, Herba Artemisiae Scopariae acts as the monarch drug and plays its core function on clearing away heat, eliminating dampness, relieving jaundice symptom, and curing hepatic injury and anti-inflammatory [14, 15]; Fructus Gardeniae as the minister drug exerts its pivotal role on accelerating the efficacy of monarch drug to purge fire, eliminate dampness, cool blood, inhibit inflammatory response, and protect against hypertension [16, 17]; Rheum rhabarbarum is widely utilized in clinic for its therapeutic action against cholelithiasis and chronic kidney disease [18]. And the drug was famous for the assistant and servant laxative agent which can have cholagogic effect, promoting diuresis, relieving jaundice, removing blood stasis, and harmonizing qi and blood. In this prescription, three ingredients exhibit synergistic properties to minimize adverse effects and enhance therapeutic outcomes [19]. Modern pharmacologic researches indicated that taking YCHD was an invasive but valid therapeutic treatment for liver disorders, cholestasis, and jaundice in China and Japan [20, 21].

Moreover, genetic predisposition of cholesterol gallstone was generally reported [22, 23]. LXR is a nuclear receptor that primarily acts to protect hepatocytes against the effects of elevated bile acids, whose agonists also have triglyceride-lowering properties and could be useful in treating certain types of dyslipidemia. LXR modulators or antagonists could potentially lower LDL cholesterol levels and even modulate high-density lipoprotein metabolism [24–26]. HMGCR acts as the rate-limiting enzyme in cholesterol synthesis and the primary site of feedback regulation in the biosynthesis of cholesterol [27]. Likewise, CYP7A1 and CYP7B1 mediate the transformation of cholesterol into bile acids in vitro [28, 29]. ABCG5, ABCG8, and ABCB4, belonging to the ATP-binding cassette (ABC) transporter family, are essential for the secretion of phospholipids from hepatocytes into bile and that disruption of these genes increases dramatically the responsiveness of plasma and hepatic cholesterol levels to changes in dietary cholesterol content [30–34]. Furthermore, PPAR-a exerts regulatory control over the expression of numerous genes encoding proteins involved in lipid oxidation and transport [35]. SREBP-2 is the primary transcriptional regulator of cholesterol biosynthesis in vivo. Deletion of Srebf-2 in hepatocytes reduced the expression of all cholesterol biosynthetic genes and rates of hepatic cholesterol synthesis [36, 37]. Thus, totally 9 genes were thought to be strongly related to the gallstone formation and can be testes though the RT-PCR techniques.

In this study, we attempted to clarify the alterations of lipid genes related to lipid metabolism that emerged towards gallstone disease and evaluate how YCHD exerted underlying influence on the transcriptions of lipid genes. The study will help to reveal the genetic relations to the potential human cholelithiasis and predict the possible functions of YCHD, which may guide the future therapy of cholesterol gallstone and satisfy the requirement of preventive treatment in modern medicine.

## 2. Materials and Methods

### 2.1. Experiment Animals and Treatments

C57BL/6J mice (male, 8-week-old, specific-pathogen-free grade) weighing 18 20g were purchased from Beijing HFK Bio-Technology Co. Ltd., China, and housed in Experimental Animal Center of Tianjin Nankai Hospital with environmentally controlled conditions. After acclimation for 2 weeks, 50 mice were randomized into 5 experimental groups: 0W, 4W, 8W, NS, and YCHD, of which 0W group was considered as control group, 8W and NS were model groups. Mice were given LD for 0, 4, or 8 weeks with isometrically intragastric administration of NS or YCHD. Food and water were ad libitum during the experimental period.

LD was abundant of 80.25% standard laboratory food, 0.5% cholic acid, 16% butter, 2% corn oil, and 1.25% cholesterol (Beijing Solarbio Science & Technology, China). YCHD was diluted into suspension at the dose of 1.54 g/kg.d (7.5 g/60kg body weight in adults daily) and supplied by Beijing Tcmages Pharmaceutical Co., LTD [38]. Experiment procedures were approved by the Animal Care and Use Committee in Tianjin Nankai Hospital, Tianjin Medical University, China (TMUh-MEC2012019).

### 2.2. Samples Collection

After an overnight fasting, but free access to water, mice were anesthetized with 4% chloral hydrate by intraperitoneal injection and then exsanguinated from retroorbital vein. Immediately after preparation, the abdominal cavity was cut along the medial line, liver tissues were removed for RT-PCR detection and histological investigation, bile were aspirated from gallbladder, and gallstone samples were obtained by cutting gallbladder. Finally, bile, gallstone, and portions of liver tissues were kept in -80°C for subsequent analysis.

### 2.3. Measurement of Bile Lipids

Bile samples were diluted appropriately with NS at the ratio of 1:7 and centrifuged at 3500rpm for 10 minutes to measure the concentrations of bile cholesterol (BC), phospholipids (PL), and total bile acid (TBA) using automatic biochemical analyzer (Cobas 8000, Germany) in accordance with standard experimental protocol. CSI was calculated to evaluate bile lithogenicity using critical Carey tables [39].

### 2.4. Histological Examination of Liver Tissues

Liver tissues were excised and fixed in 4% formalin for 2 weeks. Afterwards, fixed tissues were dehydrated, paraffin-embedded, cut into thick slices, and stained with hematoxylin eosin (HE) so as to observe histological changes under the microscope.

### 2.5. Quantitative RT-PCR for Lipid Genes

Liver tissues weighing 15mg were utilized to harvest total RNA using RNAprep Pure Tissue Kit (Tiangen, Beijing, China). After being diluted with 50*μ*l RNase-free water, RNA was quantified for purity and concentration. Subsequently, first strand cDNA derived from reverse transcription reaction was synthesized with 2*μ*g RNA and Oligo (dT)_18_ primer using RevertAid™ First Strand cDNA Synthesis Kit (Thermo, USA). Fluorescent quantitative analysis was carried out on Bio-Rad IQ5 using GoTaq® qPCR Master Mix (Promega, USA). Glyceraldehyde-3-phosphate dehydrogenase (GAPDH) was the normalizing control gene, and relative gene expression was determined by comparative 2^−ΔΔCt^ method. All procedures were according to the manufacturer's reagents and instructions. The forward and reverse primer sequences were given below: mouse liver X receptor (LXR), 5′-AGACGTCACGGAGGTACAAC-3′ and 5′-AGCAGAGCAAACTCAGCATC-3′; adenosine triphosphate-binding cassette subfamily B member 4 (ABCB4), 5′-GATCAGTGCTCTTAGATGG-3′ and 5′-ATAGGCGATGTTCTCTG-3′; adenosine triphosphate-binding cassette subfamily G member 5 (ABCG5), 5′-GGAGAACATTGAAAGAGCAC-3′ and 5′-GTTACTCGCCTCAGCAG-3′; ABCG8, 5′-GACAGCTTCACAGCCCACAA-3′ and 5′-GCCTGAAGATGTCAGAGCGA-3′; 3-hydroxy-3-methylglutaryl coenzyme A reductase (HMGCR), 5′-GGGCCCCACATTCACTCTT-3′ and 5′-GCCGAAGCAGCACATGATCT-3′; peroxisome proliferator activated receptor alpha (PPAR-*α*), 5′-TGGTTGAATCGTGAGGAACA-3′ and 5′-ATCGCCACTAAGGTGTCAGG-3′; cholesterol 7-*α* hydroxylase (CYP7A1), 5′-CTTCATCACAAACTCCCTGTC-3′ and 5′-GTCCAAATGCCTTCGCAG-3′; oxysterol 7-*α* hydroxylase (CYP7B1), 5′-CCGATTCTGCCGTCTCCTT-3′ and 5′-CCAGCCTTACTCTGCAAAGCTT-3′; sterol regulatory element-binding protein 2 (SREBP2), 5′-GCGTTCTGGAGACCATGGA-3′ and 5′-ACAAAGTTGCTCTGAAAACAAATCA-3′; GAPDH, 5′-AGGTCGGTGTGAACGGATTTG-3′ and 5′-TGTAGACCATGTAGTTGAGGTCA-3′.

### 2.6. Statistical Analysis

Statistical data were represented as mean ± standard deviation (SD), and comparisons among groups were analyzed using one-way analysis of variance with Dunnett's and Student-Newman-Keuls two-tailed tests. Fisher's exact test was used to assess enumeration data to determine whether there was an association between experimental groups. SPSS 17.0 and GraphPad Prism 6.0 were applied to delineate graphs and statistical analysis. Difference was considered as statistical level when *p* < 0.05.

## 3. Results

### 3.1. Animal Conditions

**Figure 1:**
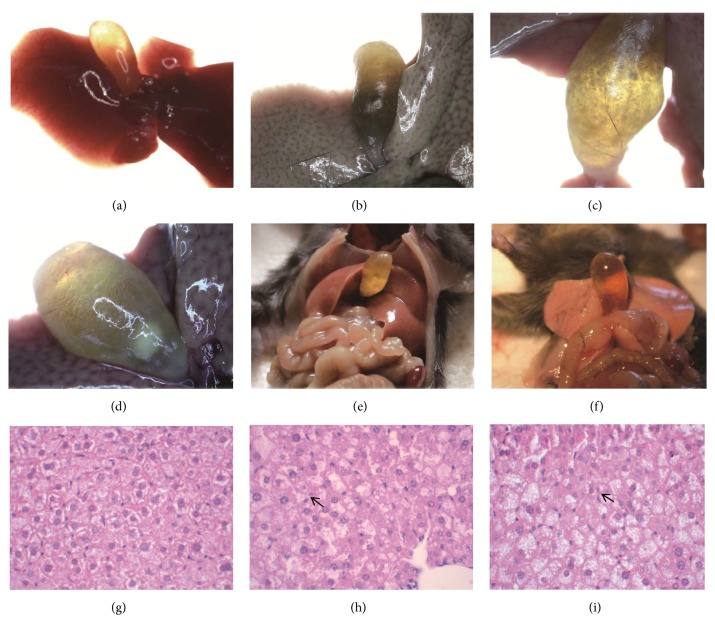
The gallbladder conditions and hepatic histological sections of different groups. (a)–(d) Analysis of gallstone evolution under 2× stereo microscope: (a) dark red liver, small and transparent gallbladder without gallstone; (b) cholestasis with greasy, fragile, light gray liver; (c) sludge or floccules in the bile; (d) massive, granulated, and white gallstones. (e)-(f) Analysis of cholesterol gallstone evolution with naked eye: (e) dense clumps of stones; (f) cholecystectasia. (g)-(i) HE staining of hepatic tissues under 400× microscope: (g) normal hepatocytes; (h) serious liver injury with fat droplets in loose cytoplasm extensively. Fatty vacuoles were filled with cytoplasm. Cells may exist with nuclear fragmentation, interstitial and double nuclear (arrow); (i) light liver injury with small vacuoles degeneration. The nucleus to cytoplasm ratio is slightly abnormal and some of the nucleus was loose and squeezed to the edge (arrow). HE: hematoxylin eosin.

Mice in 0W group behaved normally with favorable mental state and physical agility. With prolonged LD feeding, a series of abnormal symptoms like hypokinesia, inactivated state, weight loss, slow growth, and even jaundice on legs began to appear gradually, which were similar to the reported results. We observed that there were dark red liver and small and transparent gallbladder at 0W group by gross appearance and 2× stereo microscope (OLYMPUS, Japan), as shown in Figure 1(a). Figures 1(b) and 1(f) showed that cholecystectasia or cholestasis came into being and white or light gray liver became greasy, fragility with jagged edge, which were prevalent in 4W or 8W group. Moreover, sludge, crystal, or floccules as an intermediate state between gallstone and nongallstone were formed at 4W and YCHD group (Figure 1(c)).

### 3.2. Analysis of Gallstones and Effect of YCHD on Cholesterol Gallstone Evolution

With prolonged feeding time of LD, the gallstone rate was 0 in 0W group and gradually increased to 50% in 4W group; mice in 8W group possessed 100% of gallstone rate. Figures 1(d) and 1(e) showed that massive, white, or light yellow gallstone particles can be visible appreciably through the gallbladder wall, and dense clumps of stones were inspected by microscope, all of which can be regarded as the criteria for gallstones.

The gallstone rate of YCHD group (40%) was significantly less than that of NS group (100%), *χ*^2^ = 22.495, *p* < 0.001. Comparisons among 5 groups were of significance, *χ*^2^ = 31.168, *p* < 0.001; see details in Table 1. As expected, administration of YCHD displayed remarkable property in inhibiting the development of gallstone.

In addition, collected gallstones were washed and dried to perform qualitative analysis with Fourier transform infrared spectroscopy, and strong absorption peak of cholesterol gallstone was detected.

### 3.3. Histological Analysis of Liver Tissues and Effect of YCHD on Liver

Here we conducted histological analysis on mice liver tissues microscopically.  Figure 1(g) indicated that there were no abnormal changes in 0W group, while owing to the supplement of LD, serious fatty degeneration of liver and suspicious alterations of nucleo-cytoplasmic ratio happened to 8W and NS groups, which could be recognized by fat droplets full in loose cytoplasm extensively or cell nucleus close to membrane in conjunction with nuclear fragmentation or gap between nucleuses (Figure 1(h)). Histological changes in 4W and YCHD groups attenuated that situation to be small vacuoles degeneration in cytoplasm and mild liver injury (Figure 1(i)). Consequently, YCHD may play hepatoprotective role in reversing the deteriorative symptoms of liver.

### 3.4. Effect of LD on Biliary Lipid Profiles at 0, 4, 8 Weeks

**Figure 2:**
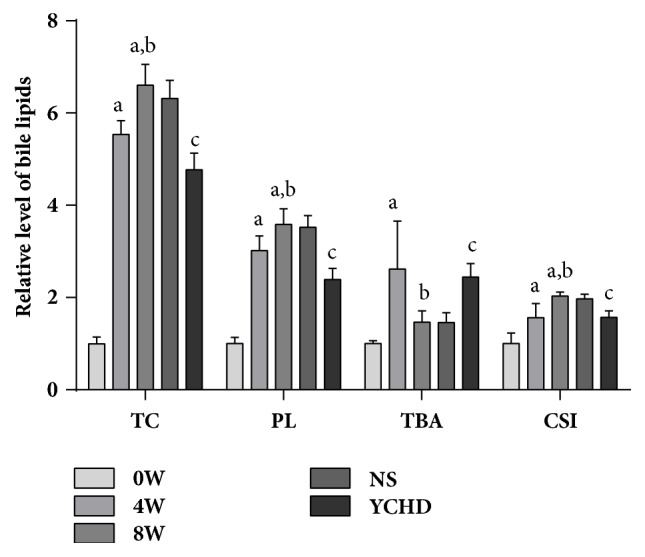
Relative levels of biliary lipid profiles and CSI (n = 7). The mean of lipid profiles and CSI in 0W was set to 1 and all values were according to mean ± SD. “a” *p* < 0.001 versus 0W group; “b” *p* < 0.01 versus 4W group; “c” *p* < 0.001 versus NS group. W: weeks; NS: normal saline; YCHD: Yinchenhao Decoction; CSI: cholesterol saturation index; BC: bile cholesterol; PL: phospholipids; TBA: total bile acid.

The relative contents of biliary lipid profiles and CSI were depicted in Figure 2. With prolonged feeding time, bile lipids and CSI displayed an increasing trend in mice groups except YCHD group. The secretion of BC in 4W (9.30±0.50 mmol/L) and 8W (11.10±0.75 mmol/L) had an approximately 5-fold increasing in comparison with that in 0W (1.68±0.24 mmol/L). PL concentration in 4W (29.51±3.15 mmol/L) and 8W (35.06±3.32 mmol/L) was 3-fold of 0W (9.78±1.34 mmol/L) as well. The severity of CSI was increased by 0.63 in 0W, 0.98 in 4W, and 1.28 in 8W group. Interestingly, a peak secretion of TBA was remarkably more elevated at 4W (125.19±49.88 mmol/L) than that at 0W (47.85±3.15 mmol/L) and 8W (69.89±11.90 mmol/L).

### 3.5. Effect of YCHD on Bile Lipids and CSI

Most significantly, our data indicated that YCHD had a certain reduction of the contents of BC (8.01±0.60 mmol/L), PL (23.35±2.34 mmol/L), and CSI (0.99±0.09) when compared with that of NS group. TBA (117.03±13.80 mmol/L) in YCHD group reached a higher content than that in NS group. YCHD may ameliorate bile lithogenicity by influencing the levels of bile lipids.

### 3.6. Effect of LD on Hepatic Gene Expression at 0, 4, 8 Weeks

**Figure 3:**
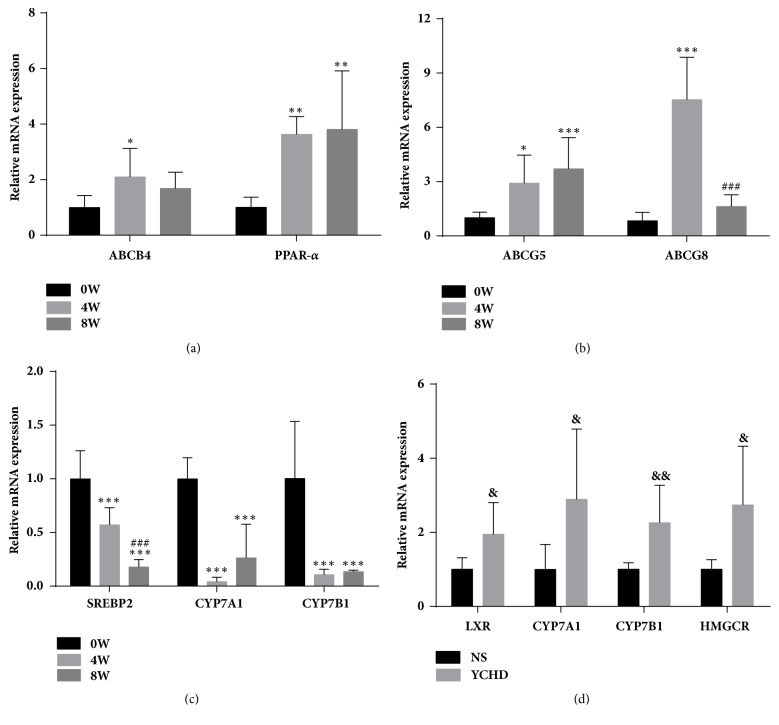
Relative expressions of hepatic lipid genes on mice cholesterol gallstone. (a)–(c) Expressing lipid gene transcriptions in 0W, 4W, and 8W (n = 6-8); (d) Expressing lipid gene transcriptions in NS and YCHD (n = 7-9). GAPDH was as normalizing control gene and all values were according to mean ± SD. “*∗*” *p* < 0.05 versus 0W group; “#” *p* < 0.05 versus 4W group; “&” *p* < 0.05 versus NS group; “*∗∗*, ##, &&” *p* < 0.01; “*∗∗∗*, ###, &&&” *p* < 0.001. W: weeks; NS: normal saline; GAPDH: glyceraldehydes-3-phosphate dehydrogenase; YCHD: Yinchenhao Decoction.

In Figure 3, the relative expressions of hepatic genes were detected using RT-PCR technique. ABCG5 and PPAR-*α* of 4W and 8W were observed to have higher transcriptional activities by approximately 3 times than that of 0W, while no significant difference but a slight elevating change happened in LXR (data not shown). Of special note, a peak expression of ABCG8 was recognized in 4W and was drastically 7-fold higher than that of 0W and 8W. Similarly, ABCB4 received a 2-fold higher transcription in 4W than that in 0W and 8W. By contrast, transrepression of CYP7A1 and CYP7B1 was appreciably found in 4W, the levels of which were lower than those in 0W and 8W. Additionally, there was a notably transcriptional repression of SREBP2 in 4W and 8W. As described previously, administration of LD in different periods may give rise to complex alterations of gene expressions, in turn generating cholesterol gallstone.

### 3.7. Effect of YCHD on Hepatic Gene Expression

Meanwhile, we found that LXR, HMGCR, CYP7A1, and CYP7B1 mRNA in YCHD group possessed approximately 3-fold increasing levels in contrast to NS group. Therefore, YCHD may effectively retard the process of cholesterol gallstone formation via regulating the activities of these genes.

## 4. Discussion

In our preliminary experiment, 10 male C57BL/6J mice vulnerable to gallstone were fed on LD for 8 successive weeks to affirmatively make mice model of cholesterol gallstone. As experiment progressed, the gallstone incidence showed an increasing tendency at 0W, 4W, and 8W. To our knowledge, mice model in vitro mimicking the human cholelithiasis was widely induced in current investigations [40]. Increasing evidence suggested that diet as a risk factor was predisposed towards cholesterol gallstone, which was consistent with the perspective of LD (high cholesterol and high fat) in the study [41]. Furthermore, the presence of high cholic acid originating from LD had potentially hepatotoxic effects on serious liver injury, and the extent of which was substantially exacerbated with the prolonged feeding time [42]. YCHD treatment may decrease the gallstone incidence and nourish the liver to reverse liver deterioration to a certain extent.

In the present study, suspicious alterations in bile lipids and gene expressions referring to lipids metabolism were mentioned. In the lithogenic state, there were two major pathways concerned with higher BC level, one of which was an input pathway to synthesize BC and the alternative was an output pathway to transfer it into bile acid. Basically, HMGCR acted as the rate-limiting enzyme to catalyze endogenous cholesterol synthesis, and SREBP-2 may activate HMGCR mRNA to perform the synthesis and uptake of cholesterol [43]. What is more, ABCG5 and ABCG8 universally heterodimerized as hepatic lipid transporters of canalicular membrane participated in cholesterol reverse transport to transport redundant cholesterol from liver to bile, and their transcriptional activation was predominantly modulated by LXR [44]. Long-term administration of LD induced plentiful dietary cholesterol and oxysterols, in part, stimulating the transcriptional activities of LXR and HMGCR. Simultaneously, the upregulation of HMGCR mRNA may be closely activated by higher SREBP-2. Nevertheless, we did not find out significant difference of LXR and HMGCR mRNA but slight increasing trend on mice groups expect YCHD group. We were not reluctant to exclude the possibility that higher expressions of ABCG5/8 may be caused by LXR or influenced by other undetected genes beyond our limited experiment. Dramatic decrease of ABCG8 from 4 to 8 weeks was probably due to the deleterious damage of canalicular membrane. However, YCHD treatment may exert a potential increasing property of LXR and HMGCR mRNA to diminish the synthesis of BC.

When it comes to the output pathway, a part of cholesterol passed through this major route to be converted into bile acid, thus leading to higher TBA. The dominant reason why BC was still higher was probably that more cholesterol was synthesized in the input pathway than that eliminated in output pathway. It should be noted that TBA concentration was up to the peak in 4W. Our explanation hypothesized that the mechanism of bile acid synthesis was blocked seriously along with progressed experiment time, and so did the enterohepatic circulation of bile acid. CYP7A1 and CYP7B1 as major enzymes of classic and alternative pathway played critical roles in catalyzing bile acid metabolism to eliminate cholesterol, and their possible regulation was under the control of nuclear receptors, such as farnesoid X receptor (FXR), LXR, and PPAR-*α* [45]. FXR as bile acid receptor was sensitive to higher bile acid to suppress CYP7A1 expression, and activation of LXR triggered by oxysterols may upregulate CYP7A1 [46, 47]. PPAR-*α* was involved in fatty acid metabolism but the effect of whether there was a relationship between PPAR-*α* and CYP7A1 remained controversial [48, 49]. With prolonged administration of LD, PPAR-*α* yielded an increasing activity, while FXR was not significantly different among 0W, 4W, and 8W groups. Another important finding indicated that the minimum expressions of CYP7A1 and CYP7B1 mRNA were recorded in 4W. In this regard, we speculated the possibility that FXR, LXR, and PPAR-*α* may impose different effects on CYP7A1 and CYP7B1, the alterations of which were therefore dedicated to the fluctuation of TBA levels. We did not rule out the possibility that consuming LD may impair hepatic function and destroy the expressions of CYP7A1 and CYP7B1. Our results also demonstrated that YCHD may improve CYP7A1 and CYP7B1 at transcriptional levels to enhance the synthesis of bile acid, thus upregulating the TBA level.

With respect to ABCB4, a lipid transporter translocated the activity of PL, preferentially phosphatidylcholine to drive PL efflux across canalicular membrane into bile [31]. Smith et al. demonstrated that higher ABCB4 may cause higher PL secretion coupled with higher cholesterol [50], which was in favor of our experiment data with higher ABCB4 and persistent increasing PL at 4W and 8W. Strikingly, ABCB4 was upregulated at mice groups of 4W and 8W but with a slight decrease at 8W. As previously mentioned, we supported the hypothesis that long-term LD may impair the canalicular membrane of liver and injure the function of ABCB4 properly.

Under normal conditions, bile lipids were mainly composed of BC, bile salt, and PL. PL may solubilise accumulated BC into harmless vesicles to solution, in turn ruling out of the body, and bile salt may facilitate the absorption of fat; the levels of three components maintained relative homeostasis. Once the balance was disrupted, compositional alterations may experience the process of cholesterol aggregation, super-saturation, and crystal and eventually progress to cholesterol gallstone [51]. In our study, dramatic alterations of BC, bile salt, and PL ultimately generated higher CSI, lithogenic bile, and cholesterol gallstone. YCHD may ameliorate bile lithogenicity (CSI<1) by lower BC and PL and higher TBA.

So far, our study was the first to address the variations of lipid genes related to the disorder of lipid metabolism during the lithogenic time at 0, 4, and 8 weeks and how YCHD possessed preventive effects on the transcriptions of lipid genes under lithogenic state. Here deficiencies still existed as our study was limited to the gene expressions of hepatic tissue, in spite of the same genes or undetected target genes localized on intestine, gallbladder, and so on. Our future considerations are required to detect the protein levels of the mentioned genes and to further confirm whether our findings in vitro had potential application towards human cholelithiasis.

## 5. Conclusions

In summary, abnormal expressions of ABCG5, ABCG8, ABCB4, PPAR-*α*, SREBP2, CYP7A1, CYP7B1 mRNA, disorders of bile lipids, and impaired hepatic changes made a significant contribution to cholesterol gallstone formation. CYP7A1, CYP7B1, LXR, and HMGCR mRNA may be efficient targets of YCHD. YCHD treatment may be a promising strategy to nourish the liver, normalize bile lipids, and attenuate gallstone formation.

## Figures and Tables

**Table 1:** Analysis of gallstone rates on 5 mice groups (n = 10).

Group	Number of mice	Number of gallstones	Gallstone rate (%)
0W	10	0	0
4W	10	5	50
8W	10	10	100
NS	10	10	100
YCHD	10	4	40^*∗∗∗*^

Significant comparisons among 5 groups were of statistical importance, *χ*^2^ = 31.168, *p*<0.001. *∗∗∗p* < 0.001 vs NS group. W: weeks; YCHD: Yinchenhao Decoction; NS: normal saline.

## Data Availability

The data used to support the findings of this study are available from the corresponding author upon request.

## Abbreviations

YCHD: Yinchenhao Decoction
LD: Lithogenic diet
W: Weeks
NS: Normal saline
CSI: Cholesterol saturation index
RT-PCR: Real-time polymerase chain reaction
BC: Bile cholesterol
PL: Phospholipids
TBA: Total bile acid
TCM: Traditional Chinese medicine
LDG: Lidan Granule
HPA: Hypothalamic-pituitary-adrenal
GAPDH: Glyceraldehyde-3-phosphate dehydrogenase
FXR: Farnesoid X receptor
LXR: Liver X receptor
ABCB4: Adenosine triphosphate-binding cassette subfamily B member 4
ABCG5: Adenosine triphosphate-binding cassette subfamily G member 5
HMGCR: 3-Hydroxy-3-methylglutaryl coenzyme A reductase
CYP7A1: Cholesterol 7-*α* hydroxylase
CYP7B1: Oxysterol 7-*α* hydroxylase
SREBP2: Sterol regulatory element-binding protein 2
PPAR-*α*: Peroxisome proliferator activated receptor alpha
SD: Standard deviation
HE: Hematoxylin eosin.
